# Imaging of psoriatic nail disease pre and post anti-TNF therapy shows persistent subclinical inflammation despite good clinical response

**DOI:** 10.1186/1471-2474-14-S1-A7

**Published:** 2013-02-14

**Authors:** Zoe Ash, Richard Hodgson, Andrew Grainger, Sibel Z Aydin, Concepcion Castillo-Gallego, Ai Lyn Tan, Helena Marzo-Ortega, Dennis McGonagle

**Affiliations:** 1 Section of Musculoskeletal Disease, Leeds Institute of Molecular Medicine, University of Leeds and NIHR Leeds Musculoskeletal Biomedical Research Unit, Leeds Teaching Hospitals, Leeds, UK

## Background

In psoriatic arthritis (PsA), nail disease is intimately linked to the presence of DIP joint enthesitis and arthritis. Nail disease has been shown in clinical trials to respond to treatment with TNF inhibitors. We used high resolution MRI to assess whether improvements in psoriasis-related nail disease correlated to improvements in associated DIP joint enthesitis, osteitis and synovitis during TNF inhibitor therapy.

## Methods

Nine patients with psoriatic nail disease in addition to active skin or joint involvement were recruited prior to the commencement of TNF inhibitor therapy. All patients were TNF inhibitor naive at baseline. Assessments at baseline and six months included: clinical assessment, ultrasound of the nails and peripheral entheses and high resolution MRI scanning of one finger. Peripheral ultrasound enthesopathy was scored according to inflammatory and chronic findings, with the sonographer blinded to disease state. DIP joint MRI scans were scored by blinded paired analysis by two musculoskeletal radiologists.

## Results

The mean patient age was 39, 2/9 patients were female. Seven patients had co-existent PsA, of whom all had DIP involvement. TNF prescription was made according to NICE guidelines. Five patients received etanercept and four adalimumab. One psoriasis patient was withdrawn at five months due to skin non-response. The results are presented here for the eight patients who completed the study.

Marked improvements were seen in clinical parameters (skin, joints and nails) as well as peripheral entheseal ultrasound assessments (Table 1). Baseline MRI scans showed DIP enthesitis, osteitis or synovitis in all PsA patients. Follow-up MRI scans showed persistent inflammatory changes in the DIP joint, distal phalanx and the soft tissues around the nail.

**Table 1:** 

		Baseline	Six months
Clinical assessment	PASI	5.25	0.76
	mNAPSI	26	7
	Swollen joint count	11	1
	Tender joint count	11	1
	
	SPARCC enthesitis index	3.1	0.4

Peripheral entheseal ultrasound assessment	Total peripheral enthesopathy ultrasound score	27.8	21.6
	
	Inflammation score related to power Doppler changes	1.0	0.1
	
	Chronic enthesopathy score	11.8	9.9

Clinical and ultrasound assessments, all reported as mean. Arthritis outcome measures reported for PsA patients only.

## Conclusions

In keeping with RCT data, a good clinical response for arthritis, skin and nail disease was seen with anti-TNF therapy in patients with psoriatic nail disease. This exploratory imaging study however shows persistent ongoing subclinical inflammation (osteitis, synovitis and enthesopathy) at six months. This could relate to either a time lag in imaging improvement or the sensitivity of the imaging techniques used.

